# Patient choice, Internet based information sources, and perceptions of health care: Evidence from Sweden using survey data from 2010 and 2013

**DOI:** 10.1186/s12913-016-1581-5

**Published:** 2016-08-01

**Authors:** Emma Wahlstedt, Björn Ekman

**Affiliations:** Social Medicine and Global Health (SMGH), Lund University, Lund, Sweden

**Keywords:** Patient choice, Internet, Health care reform, Patient satisfaction, Access to care, Sweden

## Abstract

**Background:**

Several countries have increased patients’ abilities to choose their health care providers, frequently under the assumption that patients are themselves the best agents to make such decisions. In parallel, national and regional health authorities have enhanced access to Internet based information sources (IBIS) to assist patients in making an informed choice. Relatively little, however, is known about the effect that the use of such sources has on key outcomes, including patients’ perceptions of care. The aim of this study is to analyze the role of the Internet for patients’ confidence in the provider and perceived access to care in the context of choice based reforms in Sweden.

**Methods:**

The study uses a regional, population health survey from the southern part of Sweden. Non-parametric chi-square tests are used to assess the nature of Internet users, including their gender, age and socioeconomic status. Logistic regression models are specified to analyze the role of IBIS on patient perceptions of care while controlling for other factors.

**Results:**

Actual use of Internet based information sources for health care was relatively low in 2010 and only somewhat higher in 2013. The characteristics of IBIS users varied significantly across different population groups, such that they were younger, more educated, female, and also considered themselves to be in better health compared with those who reported not using this source of health care information. Finally, the average IBIS user was less likely to report having a high level of satisfaction with respect to their primary care use; OR 0.69 [95 % CI: 0.54–0,87] and OR 0.52 [95 % CI: 0,41–0,66], for confidence in provider and perceived access to care, respectively, in 2013.

**Conclusions:**

Despite health agencies’ attempts to make information on health care providers available on the Internet, this source of health care information is not used to any large extent in the current sample. The fact that some people use this source of information more compared with others suggests the need to consider alternative ways of informing the general public about choice options. The use of Internet based sources may also be linked with the experience of actually using health services, which suggests a need to further analyze this complex consumer behavior process.

## Background

Over the past decades and in line with many other countries, several Swedish regional health authorities have introduced reform initiatives with the aim of expanding citizens’ freedom of choice in health care utilization. In particular, starting in 2007, a small number of county councils (or Regions; the administrative government units charged with providing health services to the Swedish population) introduced the possibility to make an active choice of one’s primary health care (PHC) provider [1, 2]. Partly based on these initiatives and experiences, the central government passed a national law, effective as of 2010 – the Freedom of Choice Act (LOV) – mandating all 21 county councils/Regions to, among other things, allow citizens to choose their PHC provider [3].^1^ The LOV act builds on a fairly long process of trying to reform the traditional Swedish national health service-type of system and introduce more market based elements in the provision of health care [4, 5]. The overall objective of these initiatives has been to increase the efficiency of the health system while maintaining its main focus of equal access to health services [6]. The short-term impacts of these reforms on patient access and use of health services have been found to be largely positive in recent analyses [7–10].

An important aspect of the choice-based reforms in Europe and elsewhere is that adequate and complete information is available for consumers to be able to make an informed choice. While such information can come from several different sources, the Internet is an increasingly more important source of health information, including for the ability to make a provider choice [11–13].

Consequently, in parallel with the Swedish health reforms, several initiatives have been made to increase accessibility of health care related information, not least over the Internet. For example, in 2006, a national initiative was taken involving the National Board of Health and Welfare (Socialstyrelsen) and the National Association of Swedish Counties and Municipalities (SKL) to set up a web-based platform, *“Open comparisons”* (OC; *Öppna Jämförelser*), to be able to compare the quality and efficiency of public services across counties. The OC system presents aggregate data on a range of health care indicators and is mostly aimed at the counties themselves in their planning and development work.

To enable citizens to make an informed provider choice, similar Internet based systems have been developed over the past few years. The purpose of these systems is to enable the general public to compare individual providers (at the clinic or unit level, not the individual GP level) across a set of “quality indicators” and, moreover, to make the actual choice of PHC provider. Examples of these systems include www.1177.se (The Health Care Guide; *Vårdguiden*, run by the SKL), www.varden.se (a privately run Internet service), and www.omvard.se (also a private initiative). Similar web-based information platforms exist in most other European countries, such as the NHS Choices in the UK (http://www.nhs.uk/Pages/HomePage.aspx) and the Danish *Sundhed* (Health; https://www.sundhed.dk/).

There are, however, several issues associated with providing citizens with health information over the Internet, both with respect to effectiveness and distribution. First, the information needs to be easily understood by laypersons. In the case of Sweden, the regulations require that health care related information, regardless of its source, is “accurate, relevant, comparable, easily understood, and easily accessed” [14, page 7]. Presenting health related information, including the comparison of individual providers, has, however, been shown to be difficult [14–16]. And, second, from a distributional perspective, not all people have the same access to and skills in using the Internet [17, 18]. The evidence base for the extent to which these sources of provider information is used and whether it affects patient experiences is currently limited [19, 20]. While it has been found that patient satisfaction is partly determined by institutional and context factors (like waiting times, geographic access, and provider friendliness) [21, 22], less is known about the actual use of Internet based information sources (IBIS) such as those identified above and patients’ experiences of health care [23].

The aim of this study is to contribute to improving the understanding of the role of the Internet for patients’ perceptions of health care in a context of choice-based reforms. In particular, the objectives of the study are to assess the nature of Internet users in terms of prevalence and their characteristics, including by gender, age and socioeconomic status, and to analyze the association between the use of the Internet for health care information and key outcomes, including patient confidence in providers and perceived access to health care.

The study is set in the southern region of Skåne, which has a population of around 1,2 million people, or 13 % of the total population of Sweden. Skåne was one of the first regions to commence the implementation of the patient choice act of 2010 [7]. In line with most other regions of the country, Skåne had initiated a series of changes through most of the 2000s that partly included increased patient choice and the establishment of private providers. These changes were partially a response to indications that the health care in Skåne was not delivered efficiently and that the general public was dissatisfied with the services [24].

## Data and methods

### Data

This study utilizes data obtained from the Skåne section of the national population survey *Vårdbarometern* (the VB-survey henceforth) from the years 2010 and 2013.^2^ This survey has been implemented since 2007 (with broader participation since 2010) and collects individual level data across five themes: *Contact with health care*; *Attitudes toward different treatment options*; *Access to health care*; *Confidence in health care*; and *Financing and priorities of the health care*.^3^ In addition, a number of background, individual level information is collected, such as age, gender, and level of education. Of relevance to the objectives of this study, the survey contains information on perceived confidence in providers, knowledge of health care, and use of the Internet in learning about providers and health care options. Access to the anonymized data and permission for its use were obtained through the regional supervisor of the survey for Region Skåne.

In 2010 the VB-survey revised its questionnaire to make it a survey purely focused on attitudes and behaviors surrounding health and health care, excluding previous patient experience questions. For the purposes of this study the 2010 survey is seen as a baseline survey given the introduction of the nation-wide PHC choice act in that year. Finally, the VB-survey contains very few missing data due to the direct interview approach. Generally, only those questions that are not relevant to the individual generates missing data (such as not having visited a hospital in the past year). For all of the variables used in the current study, the share of missing data was less than five percent leading us to keep all observations in the analytical sample.

The information used in this study was derived from the two themes *Access to health care* and *Confidence in health care*, and from background questions on participants’ health, education, age, and gender. Individuals’ reported access to health care and their confidence in primary health care providers are the main outcome variables of the study. In both 2010 and 2013, around 2/3 of the sample said their confidence in their PHC-provider is high (*How strong is your confidence in the PHC-providers of your county?*). In 2010 around 80 % of the participants agreed with the statement: *I have access to the health care I need*. In the larger sample of 2013, this share was slightly lower at 77 %. Access to care in this case was not specific for primary care but included the whole spectrum of health care providers. Since the primary care is supposed to be the first contact with health care for an individual in Sweden, it is assumed that the perceived access includes primary health care for most of the respondents [25].

The main analytical variable of the study is reported use of Internet based information sources (IBIS). This variable is derived from the question *Have you used the internet to compare caregivers during the past 6 months?*. The original three alternatives *Yes*, *No,* and *I don’t use the internet* have been combined to form one dichotomous indicator, *Have used IBIS*, which takes the value 1 for those who responded positively to that question, and 0 for the last two response options.

Individual health status and health knowledge were measured by three questions: *How do you assess your own health status?*, *Do you have any long term health problems?*, and *How good or bad would you say your health knowledge is?*. The original five category responses to these questions were transformed into three separate dichotomous variables, *Good* (coded as 1) and *Not good* (coded as 0) for each of the two health status variables and the health knowledge variable.

Demographic characteristics were represented by age and gender. The age variable was modified from the original eight groups into three age groups: *Young* (18–39), *Middle age* (40–59) and *Old* (60+). This transformation generated a well-balanced sample across these age categories. In 2010 there were more male participants than female, while in 2013 there was a slight overweight of women; see Table 1 for details.

**Table 1:** Frequencies and shares of analytical variables, 2010 and 2013

Variables		2010		2013	
	Categories	*n* (%)	Missing *n* (%)	*n* (%)	Missing *n* (%)
Confidence in PHC provider	*High*	607 (63)	41 (4,1)	3640 (63)	244 (4,1)
	*Not high*	352 (37)		2116 (37)	
Access to care	*Agree*	803 (81)	8 (0,8)	4511 (77)	143 (2,4)
	*Do not agree*	189 (19)		1346 (23)	
Use of IBIS	*Have used*	57 (6)	16 (1,6)	326 (6)	945 (15,8)
	*Have not used*	927 (94)		4729 (94)	
Self-assessed health knowledge	*Good*	876 (89)	11 (1,1)	5038 (88)	270 (4,5)
	*Not good*	113 (11)		692 (12)	
Self-assessed health	*Good*	753 (76)	10 (1)	4368 (76)	252 (4,2)
	*Not good*	237 (24)		1380 (24)	
Long-term health problem	*Yes*	360 (37)	17 (1,7)	2176 (38)	280 (4,7)
	*No*	623 (63)		3544 (59)	
Age	*Young (18–39)*	213 (21)	0 (0)	1246 (21)	0 (0)
	*Middle age (40–59)*	334 (33)		1660 (28)	
	*Old (60+)*	453 (45)		3094 (52)	
Gender	*Male*	558 (56)	0 (0)	2931 (49)	0 (0)
	*Female*	442 (44)		3069 (51)	
Education	*9 years*	166 (17)	19 (1,9)	1325 (23)	298 (5,0)
	*12 years*	437 (45)		2360 (41)	
	*<12 years*	378 (39)		2017 (36)	
Visit to care	*Yes*	642 (64)	2 (0,2)	3849 (64)	22 (0,4)
	*No*	356 (36)		2129 (36)	
N		1000		6000	

Source: Vårdbarometern Survey Skåne, Sweden, 2010 and 2013. *PHC* Primary health care

Reported level of education described the socioeconomic status. This variable was adapted from the question *What is your highest completed education?* and modified from four groups into three separate educational categories: *9 years* (primary school only), *12 years* (high-school) and *More than 12 years* (above high-school) to capture the effect of having an education commensurate with the main education levels in Sweden. The data do not contain information on individuals’ annual incomes or their wealth.

### Methods

The objectives of the study are addressed by statistical analyses of the data described above. First, to assess the use of Internet based information sources in terms of user characteristics, non-parametric chi-square tests are applied to evaluate any significant differences between the identified groups of users. Second, to analyze the association between the two main outcome variables – (i) Confidence in PHC-provider and ii) Perceived access to care – and IBIS, while controlling for other factors, the study identifies the following general regression model:

1 $$ \Pr \kern0.1em \left({\mathrm{Y}}_{\mathrm{i}}=1\right)=\alpha +{\beta}_{\mathrm{IBIS}}\mathrm{IBIS} + {\beta}_{SocDem}\kern0.1em  SocDem+{\beta}_{SocEc} SocEc+{\beta}_{Health} Health+\varepsilon $$

Based on this general model, two specific models are tested. When analyzing the effect of IBIS on provider confidence, Y takes the value 1 if the individual *i* has confidence in the provider and 0 otherwise. When analyzing the effect of IBIS on perceived access to care, Y takes the value 1 if the individual reports having access to care and 0 otherwise. The variables *SocDem* and *SocEc* are vectors of demographic (age and gender) and socioeconomic (education) indicators, respectively. In the two separate models *Health* contains three health related indicators: the individuals’ self-assessed health status, any long-term health issue, and reported health knowledge. The *betas* are variable coefficients to be estimated (reported as odds ratios) and *epsilon* captures all other factors affecting the outcome not in the models. In both cases, the models are estimated by means of logistic regression to account for the binary outcome variables. All data analysis was done using IBM SPSS v.22.

The current study and the data collection on which it is based did not involve any experiment or other invasive methods on humans. The data are fully anonymized and the identity of any of the respondents is not known to the users of the data and the use of the data does not require ethical clearance. The participants were informed that participation was voluntary and a written consent was not required [26].

## Results

### Sampling

The VB-survey has a cross sectional study design and the study population consists of individuals 18 years and older at the time of the survey (around 949,000 and 976,000 people in Skåne in 2010 and 2013, respectively). Data are collected during two separate rounds, one in the spring and one in the fall, through phone interviews where the interviewer reads the questions to the respondents and registers the responses. The sampling frame of the survey is a database that contains the telephone numbers (cell and fixed-line) of all people living in the country (except unlisted numbers) [26]. The VB-survey aims to interview a representative sample of around 0.5 % of the adult population by making a random draw from the sampling frame. This translates into a minimum of 1,000 people from a medium-sized county, i.e. the survey terminates when the target number of participants have been reached. In 2010, 1,000 persons were interviewed in Skåne and in 2013 6,000 people were interviewed. The survey response rate was similar in both years at 40.5 % and 45.9 %, respectively; i.e. slightly more than twice the number of persons were contacted for interview than the final number of participants (the interviewers make a maximum of five contact attempts and no rewards are given for participation). The response rate of the Skåne VB-survey is similar to that in other counties and to that of other comparable household surveys [26].

### Descriptive statistics

Table 1 shows the frequencies and relative share of the respective response categories for all analytical variables used in the study.

Around two-thirds of the sample in both years report that they have high confidence in their provider. A fairly stable majority also report that they do think that they have access to the care that they need. Most people are in relatively good health and have good general knowledge about their health. The largest age category in the sample is Old (over 60 years) and the majority of respondents have primary and secondary (below university) education.

### Prevalence of IBIS use and user characteristics

The actual use of the Internet to obtain health care information is relatively low in this sample of the general adult population of Skåne (Table 1). In 2010, around 5.8 % of the full sample stated that they had used an Internet based information source for this type of information; see Table 1 for details. Three years later this share had increased to around 6.4 %, an approximate 10 % increase over this period.

With respect to the demographic and socioeconomic profile of the IBIS users, Table 2 shows the comparisons analysis by observed groups for both years. Women appear to use IBIS to a larger extent than do men and younger age groups (below 60 years) reported using the Internet more than the older group. Also, more years of education appear to lead to a higher use of the Internet for obtaining health information as do having made a previous visit to a health service provider.

**Table 2:** Profiles of IBIS users 2010 and 2013

		Have used IBIS	
Variables	Categories	2010	2013
		*n* (%)	*n* (%)
Self-assessed health knowledge	*Good*	54 (95)	302 (94)
	*Not good*	3 (5)	19 (6)
Self-assessed health	*Good*	37 (66)	242 (75)
	*Not good*	19 (34)	79 (24)
Long term health problem	*Yes*	26 (46)	137 (43)
	*No*	329 (54)	180 (57)
Age	*Young (18–39)*	18 (32)	120 (37)
	*Middle age (40–59)*	29 (51)	103 (32)
	*Old (60+)*	10 (18)	103 (32)
Gender	*Male*	22 (39)	141 (35)
	*Female*	35 (61)	212 (65)
Education	*9 years*	0 (0)	30 (9)
	*12 years*	24 (43)	122 (38)
	*<12 years*	32 (57)	172 (53)
Visit to care	*Yes*	45 (79)	239 (74)
	*No*	12 (21)	85 (26)

Source: Vårdbarometern Survey Skåne, Sweden, 2010 and 2013

In 2010, self-assessed health, chronic ill health, and self-reported health knowledge do not appear to matter significantly for whether a person used the Internet or not to obtain health information. However, with the exception of self-assessed health (SAH), these factors did appear to matter in 2013. For the other factors there were no large differences in these frequencies between the two survey years.

### The use of IBIS and patient satisfaction

Turning now to the second part of the analysis, Table 3 shows the results of the logistic regressions of the two models. For both analyses (Confidence in provider, left part of Table 3 and Perceived access to care, right part, for 2010 and 2013, respectively), the key analytical variable is Internet use, *IBIS*. Across both outcomes and for both years, the odds ratio of the Internet use variable is below one, suggesting that individuals who do use the Internet to obtain health information has lower confidence in PHC-providers and report having less access to care than individuals who do not use this source of health information, holding other factors constant. For example, a person who used the Internet for information about health care in 2013 had approximately 48 % lower odds of reporting having good access to health care compared with a non-user of IBIS. With the exception of the first model in 2010, these differences are statistically significant.

**Table 3:** Logistic regression models of i) Confidence in PHC provider and ii) Perceived access to care 2010 and 2013

	i) Confidence in PHC provider				ii) Perceived access to care			
Variables	*p*-value	2010 OR [CI]	*p*-value	2013 OR [CI]	*p*-value	2010 OR [CI]	*p*-value	2013 OR [CI]
Knowledge and use of IBIS								
* No*		1		1		1		1
* Yes*	0.078	0.594 [0.332–1.061]	0.002	0.686 [0.542–0.868]	<0.001	0.291 [0.160–0.531]	<0.001	0.518 [0.405–0.663]
Age								
* Old (60+)*		1		1		1		1
* Young (18–39)*	0.008	0.594 [0.404–0.873]	<0.001	0.642 [0.548–0.753]	0.045	0.608 [0.374–0.988]	0.031	0.816 [0.679–0.981]
* Middle age (40–59)*	0.014	0.662 [0.476–0.920]	<0.001	0.713 [0.617–0.824]	0.001	0.511 [0.341–0.765]	0.001	0.762 [0.646–0.897]
Gender								
* Female*		1		1		1		1
* Male*	<0.001	1.674 [1.264–2.218]	0.014	1.163 [1.031–1.311]	0.193	1.257 [0.891–1.773]	0.686	1.029 [0.896–1.181]
Education								
* 9 years*		1		1		1		1
* 12 years*	0.288	0.793 [0.517–1.216]	0.005	0.771 [0.644–0.924]	0.31	0.750 [0.430–1.307]	0.001	0.705 [0.571–0.871]
* More than 12 years*	0.985	1.004 [0.642–1.570]	0.008	0.781 [0.650–0.938]	0.3	0.738 [0.416–1.310]	0.006	0.739 [0.597–0.916]
Self-assessed health knowledge								
* Not good*		1		1		1		1
* Good*	0.035	1.605 [1.034–2.489]	0.085	1.186 [0.977–1.143]	0.425	1.238 [0.733–2.091]	0.128	1.184 [0.953–1.470]
Self-assessed health								
* Not good*		1		1		1		1
* Good*	0.15	1.313 [0.906–1.900]	<0.001	1.409 [1.198–1.656]	0.001	2.089 [1.364–3.198]	<0.001	1.788 [1.499–2.131]
Long term health problems								
* Yes*		1		1		1		1
* No*	0.291	0.840 [0.607–1.161]	0.953	0.996 [0.868–1.143]	0.106	0.723 [0.488–1.071]	0.416	0.937 [0.801–1.096]
Have you visited healthcare?								
* No*		1		1		1		1
* Yes*	0.146	1.256 [0.924–1.708]	0.757	1.021 [0.897–1.162]	0.804	0.952 [0.644–1.407]	0.124	0.888 [0.763–1.033]
Reported Model R-squared		0.066		0.028		0.110		0.038
N		912		4792		943		4802

Source: Vårdbarometern Survey Skåne, Sweden, 2010 and 2013. 1 signifies the comparison category. *PHC* Primary health care

The effect of IBIS appear to be somewhat larger in 2013 than in 2010 for both outcomes and the models explain a slightly larger share of the variation in perceived access to care for both years.

With respect to the other factors, age would seem a relatively important aspect in explaining the observed outcomes: *Young* and *Middle age* both have lower odds of stating they have adequate access to care compared to the *Old* group (Odds ratios 0.608 [95 % CI: 0.374–0.988] and 0.511 [0.341–0.765], respectively).

For those assessing their own health as *Good*, the odds ratio of 1.788 [95 % CI: 1.499–2.131] in 2013 showed that they had higher odds of perceiving that access to care was good than those assessing their health as *Not good*. For all other independent variables entered in the model, no statistically significant relationships were established by the models.

In addition to the specifications applied above, the models were also tested using alternative definitions of some of the variables. For example, the IBIS variable was also defined to include reported knowledge of the existence of the Internet based information source, www.1177.se. While this resulted in slightly different absolute values of the beta coefficient to those in Table 3, the overall results were robust to these alternative specifications. The next section includes further sensitivity analyses.

## Discussion

The key result of this study is that, while controlling for a set of confounding factors, the use of the Internet for health care related information is found to be associated with lower odds of having high confidence in providers and of reporting having good access to needed health care. Furthermore, the analysis also finds a gradual increase in the use of the Internet in learning of health care options, albeit from a relatively low level. Finally, in this sample of the general adult population of Skåne, the IBIS user is predominantly female, younger, and more educated compared with non-users. This section discusses these main findings in more detail, starting with a discussion of the study’s main limitations.

### Methodological discussion

One limitation of the study is the nature of the data. While the samples are of acceptable size, the cross-sectional design effectively prohibits a causal analysis of the relationships. The implication of this is that it is not possible to say, from the results of this study alone, that using the Internet to obtain health information leads to less satisfaction with health care, as it could also be the case that for some reason, people who are less content with health services tend to use this source of information to a larger extent than do more generally satisfied persons [27]. One such reason could be an inherent attitude toward both the Internet as such and toward medical care [28]. Failure to include an indicator of such an attitude would render the IBIS variable endogenous in the models and its estimated coefficients would be biased and possibly inconsistent. The analytical solution to the issue of possible unobserved heterogeneity and associated endogeneity of, in this case, the IBIS variable is to apply instrumental variable (IV) estimation techniques [29]. Specifically, this would involve replacing the IBIS variable with an indicator that is associated with IBIS use, but not with individuals’ attitudes toward the Internet and health care (now present in the epsilon error term in the models). As no such information is available in the current sample data the interpretation of the results needs to be made with caution, especially the absolute sizes of the parameter coefficients. Relatedly, the deletion of all observations with incomplete data in the estimation models may also lead to some informational loss.

A second methodological concern related to the data is the way in which the variables are measured. For example, access to care and confidence in providers are measured from specific questions on these issues in the VB-survey. While this provides a transparent and straightforward way of measuring these outcomes it may limit the measures’ comparability with those of other studies that use different ways of measuring access and patient confidence [30, 31]. In addition, the re-categorization of some of the dichotomous variables used in the analyses facilitates their interpretation, but may also reduce the power of the estimates.

A final data issue relates to the sampling of participants. While the response rate is comparable with that of other similar surveys, the VB-survey aims to include a certain number of participants (1,000 and 6,000, respectively). Although this approach ensures a sufficient number of individuals in the final sample, it may also subject the survey to the element of self-selection given that participation is voluntary. Those who decline to participate may do so for certain, non-random reasons, such as having worse health or being significantly younger than those who do participate. For example, at the national level, in the 2013 survey, a total of 15,489 contacted persons declined to participate. Other reasons for non-response included *Wrong telephone number* (3,257 persons), *Deceased* (100), and *Not eligible for other reasons* (1,202) [26].

### Discussion of main findings

Notwithstanding these limitations in the data, the study’s findings are of relevance to both the scientific evidence base on the role of the Internet in individuals’ health care seeking behaviors and to policy development. The main finding of the study is that the use of Internet based information sources to obtain relevant information about choosing a service provider or learning of the quality of different providers reduces the odds of reporting general satisfaction with the health services. While patient perceptions of health care is likely to depend on many other things than the particular source of information, the effect of Internet use on various outcomes, including these ones, is important, particularly in light of the raised expectations among policy makers that this source of information is having, also in the area of health care [32, 33]. Depending on the role that the act of seeking information plays in individuals’ overall health care consumption, this may or may not be seen as counter-intuitive. If the gathering of information is seen as an integral part of the health care consumption experience, it may well be that overall dissatisfaction with health services also includes unhappiness with the information about health care as such information has been found to be both incomplete and inaccurate [15, 34, 35].

While it may be that people are displeased with the Internet as the source of information as such, rather than the actual content, it has been noted that the information available to compare caregivers – frequently various types of quality indicators and performance data – is not always what the public is looking for [11]. Furthermore, recent analyses from Sweden suggest that the demand for various types of provider and health care information varies considerably by gender, education, and health status [17, 36]. For example, those with higher education report being more interested in factors such as provider competences and ownership of clinic compared with those with lower education who say they seek information on the clinical quality of the providers [36].

Regardless of the mechanisms, the findings speak to a general need to consider carefully how health authorities and others inform their citizens about the choice of health care provider and their relative quality. In a recent review of patients’ attitudes toward internet based health information sources in the Netherlands, Hendrikx et al. [37] noted the importance of designing such sources in a clear and useful manner. In addition, international analyses stress the importance of the possibility of being able to make an informed provider choice and its effect on satisfaction with health care [11, 38, 39]. Similarly, from the perspective of patient choice policies, Curtice and Heath [21] report that people in the UK generally do want to be able to choose their provider, but also that those who are more in favor of choice are also less satisfied with the health services in the UK. These and similar findings point at the need for an improved understanding of the complexities involved in consuming health care.

One possibly important, although seemingly overlooked, factor in understanding the impact of health information and its source, on the one hand, and outcomes, such as satisfaction and confidence in provider, on the other, is the way that being more informed about one’s illness and different treatment alternatives changes the expectations that one has on treatment experiences [40, 41]. Although it is beyond the reach of this study to assess the effect of expectations, much evidence has been generated about the important role that individuals’ expectations play in understanding patient satisfaction [42, 43]. An important hypothesis worthy of further investigation is the role that pre-consultation information plays in modifying the subsequent treatment experience. Recent studies have suggested that this involves understanding the complex interplay between information, its source, and actual patient-provider interaction [44, 45].

### Use of IBIS

The finding that relatively few people appear to use the Internet as a source of health care information is an important one and is in line with those of other studies [17, 22, 46]. In the current choice-based reforms across European and other health systems, much of the information needed to make an informed choice across provider options is Internet-based. In addition, in the case of Sweden, the way individuals choose a provider differs from one county to another.^4^ For example, in the county of Skåne, people are asked to make the provider choice using two alternative methods, both of which are online-based and one which requires filling out a form (for signing and submission as hard-copy) available on the Skåne region web-page.^5^ While it may seem rational to develop web-based systems for citizens to make their provider choices, it also risks excluding those with no or reduced access to the Internet, limited skills in using such systems, and those who refrain from accessing the web-pages for other reasons.

A general observation is that as the ability to choose health care provider increases, the demand for relevant information on critical aspects of such a choice also increases. However, Glenngård et al. [46] note that making a choice does not necessarily mean switching provider, but choosing to stay with the current one. If such a “passive choice” is the result of resorting to the default option, this could decrease the perceived need for and use of information, regardless of the source. However, if this is the case, the purpose of the reform is somewhat defeated as the key underlying assumption on which the choice-reforms are based is that well-informed consumers of health care move to those providers that are of higher quality and leave those of lower quality [17]. This and other studies point to the need for further investigations in these and related matters of availability of information, patients’ actual choices, and market changes [47].

Several previous studies, both Swedish and international ones, have noted the relatively low levels of Internet use for health care information. Dixon et al. [48] show in their report on patient choice in the UK that only eight percent of patients asked had used the internet to find health information and only four percent used the government funded site “NHS choices” (at www.nhs.uk), created specifically to aid in the choice making process in that system. Furthermore, in the literature surrounding health status and information seeking behavior, it is noted that having a health problem can interfere with the information seeking process because of the energy it takes [49]. Searching for and interpreting information is such a laborious task that to willingly make the effort, there has to be a certain urgency to the situation [11, 50] and this urgency is often not associated with a planned choice of primary care provider. More generally, studies have found a certain level of “choice fatigue”, as choosing one’s PHC-provider is only one of many choices citizens are expected to make across multiple social and economic domains [36].

### Profile of users

While the finding that the use of the Internet to seek health care information is low in general is relevant in and of itself, actual users of this source of information also vary by demographic indicators and by socioeconomic status. This finding has also been reported elsewhere and is relevant to health authorities when designing and disseminating information to citizens about their rights and options to choose provider. The existing evidence suggests that the demand for and use of information sources is largely dependent on an individual’s socioeconomic status, age and health status [51]. The socioeconomic status often refers to length of education and the consensus is that having more education has a positive influence on the information seeking behavior [51, 52]. In addition, more educated persons are usually better at interpreting the available information, all of which would suggest that better educated people obtain an advantage over less educated when it comes to making an informed choice of health care.

It is also stated that the elderly are less prone to use the internet as a source for their health information needs [46, 53]. Age was a factor that provided a paradoxical relationship to information use. In terms of use of sources like the Internet, elderly were described in the literature to be less likely to use them than the younger generation. Lack of knowledge of how to work a computer and the internet as well as distrust towards information found on the Internet may create a barrier for use [17, 51]. The elderly are often described as more concerned with their health and provider options than are the young and, furthermore, the elderly are more positive towards receiving health care information. Gatto and Tak (ibid.) describe how Internet use among the older generations may change as they gradually bring computer skills from their work into retirement with them, providing a possible explanation to the increase in older IBIS users. If this trend continues, the overall number of IBIS users is likely to increase over the years as also the older generation becomes more skilled in computer related tasks.

Health status has also been proposed to influence the act of seeking information [49]. However, the way a person’s health affects her demand for health information most likely differs whether it is a more urgent episode of disease or if the person suffers from a chronic illness. It might be postulated that an urgent need for medical care would prompt an interest in learning of treatment options and possible providers and the Internet might be one source of such information. A person suffering from a chronic illness may, on the other hand, be more interested in learning of alternative treatments from other sources, such as personal communication with a health care staff or other persons with similar experiences.

One additional aspect that appears to be mostly overlooked in the data is the issue of availability of health care information in minority languages. In Sweden the information about choosing a provider is also available in about ten languages other than Swedish.^6^ However, this and other studies from Sweden [17] have not been able to shed light on the ethnic profile of Internet users. Given the relatively high shares of immigrants in many European countries, this would seem to be an issue of relevance, including from an equity perspective [54].

## Conclusions

Current efforts to reform health systems in the direction of more choice for patients and an increase in the public-private mix of providers acting on more or less competitive markets require informed patients and health consumers. The Internet is one important source of relevant health information. Health authorities and health care payers more generally need to carefully consider the extent to which all citizens have access to whatever information is made available through the Internet. Based on this and other studies, there appears to be considerable scope for improving access to relevant health care information.

Internet based information sources for health care choices may affect also important outcomes of health care, such as trust in providers and perceptions of accessibility of care. With the expected growth in the amount of information made available through this source and the often complex nature of the information more research will be needed to address both the efficiency and the distributional aspects of this key feature of modern, market-based health systems.

## Footnotes

1 In parallel, the ability of private for-profit and not-for-profit type of providers to become established has also increased. While also this aspect of recent reforms is important, it is not subject to analysis in this study.

2 See http://www.vardbarometern.nu/om-vardbarometern.aspx for details on this survey; in Swedish

3 Themes and variable names have been translated into English by the authors.

4 See http://www.1177.se/Other-languages/Engelska/ for details.

5 See http://www.1177.se/Skane/Regler-och-rattigheter/Halso--och-vardval-i-Skane/ for details.

6 See http://www.1177.se/Other-languages/, for details.
